# Silk-hydrogel Lenses for Light-emitting Diodes

**DOI:** 10.1038/s41598-017-07817-1

**Published:** 2017-08-03

**Authors:** Rustamzhon Melikov, Daniel Aaron Press, Baskaran Ganesh Kumar, Itir Bakis Dogru, Sadra Sadeghi, Mariana Chirea, İskender Yılgör, Sedat Nizamoglu

**Affiliations:** 1 Department of Electrical and Electronics Engineering, Koc University, 34450 Sariyer, Istanbul Turkey; 2 Graduate School of Biomedical Sciences and Engineering, Koc University, 34450 Sariyer, Istanbul Turkey; 3 Graduate School of Materials Science and Engineering, Koc University, 34450 Sariyer, Istanbul Turkey; 4 Department of Chemistry, Koc University, 34450 Sariyer, Istanbul Turkey

## Abstract

Today the high demand for electronics leads to massive production of waste, thus green materials based electronic devices are becoming more important for environmental protection and sustainability. The biomaterial based hydrogels are widely used in tissue engineering, but their uses in photonics are limited. In this study, silk fibroin protein in hydrogel form is explored as a bio-friendly alternative to conventional polymers for lens applications in light-emitting diodes. The concentration of silk fibroin protein and crosslinking agent had direct effects on optical properties of silk hydrogel. The spatial radiation intensity distribution was controlled via dome- and crater-type silk-hydrogel lenses. The hydrogel lens showed a light extraction efficiency over 0.95 on a warm white LED. The stability of silk hydrogel lens is enhanced approximately three-folds by using a biocompatible/biodegradable poly(ester-urethane) coating and more than three orders of magnitude by using an edible paraffin wax coating. Therefore, biomaterial lenses show promise for green optoelectronic applications.

## Introduction

Electronic device consumption increases day by day and this trend leads to massive amounts of electronic waste (e-waste). For example, in 2011 more than 2 million tons of e-waste was produced by U. S. only^1^, and even though a significant portion of e-waste (24.9%) is recycled, the remaining waste generates a substantial hazard to the environment^2^. When the global e-waste produced by the entire world is considered, the consequences become catastrophic (e.g., great garbage patch in the Pacific Ocean). Therefore, a transition toward ‘green’ materials is necessary in electronics for environmental protection and sustainability.

The invention of efficient blue light-emitting diodes (LEDs) has opened up a new way toward bright and energy-saving solid-state lighting (SSL)^3^, which has a very important potential to replace conventional light sources because of its energy saving, long lifetime and compactness^4, 5^. For example, SSL can generate 50% reduction in electricity consumption and therefore decrease carbon emissions by 28 million tons per year, if the targeted LED performance levels are reached^6^. Light-emitting diodes (LEDs) are now being used for lighting applications in cars, homes, offices and displays. According to BCC Research, the LED market had a value of $38.2 billion in 2013 and this will approximately triple by 2019^7^. As the production of LED and LED based devices will increase in the future, the same it will happen with the production of electronic waste. A typical LED chip is made of a semiconductor die, wire bonding, a heat sink, metal contacts, packaging and a lens^8^. The lens occupy a significant portion of the whole LED device and are generally fabricated from high-performance silicone based polymers and epoxy resins, which are widely available, but not biodegradable^9, 10^. Hence, a transition toward LED lenses based on eco-friendly materials has the potential to enhance the environmental protection and sustainability.

Biocompatible material based hydrogels are widely used in tissue engineering as scaffolds^11, 12^. Silk fibroin protein obtained from Bombyx-mori cocoons has been tested for various biomedical applications^4, 13–15^, and a new type of hydrogel made of silk fibroin was recently demonstrated, which was used for microfluidic systems, multiphoton micromachining and tissue engineering^16–21^. Even though liquids have been used for various optoelectronics device applications, such as liquid crystals displays (LCDs), fluidic adaptive focusing and colour conversion layers^22, 23^, silk hydrogel lenses have not been investigated for LED applications.

In this study, we explored the feasibility of using silk hydrogels as a lens material for light-emitting diodes. We investigated the optical properties of the silk hydrogel lenses and showed that the fibroin protein concentration and cross-linking have a direct effect on the optical property of hydrogels. We demonstrated high control on the spatial light distribution via dome- and crater-type silk-hydrogel lenses for LED applications. Moreover, the hydrogel lenses extracted light with high efficiency on warm white LEDs. Finally, their stability was significantly enhanced by using a polymer top coating.

## Results and Discussion

### Formation and optical transparency of silk hydrogel

**Figure 1:**
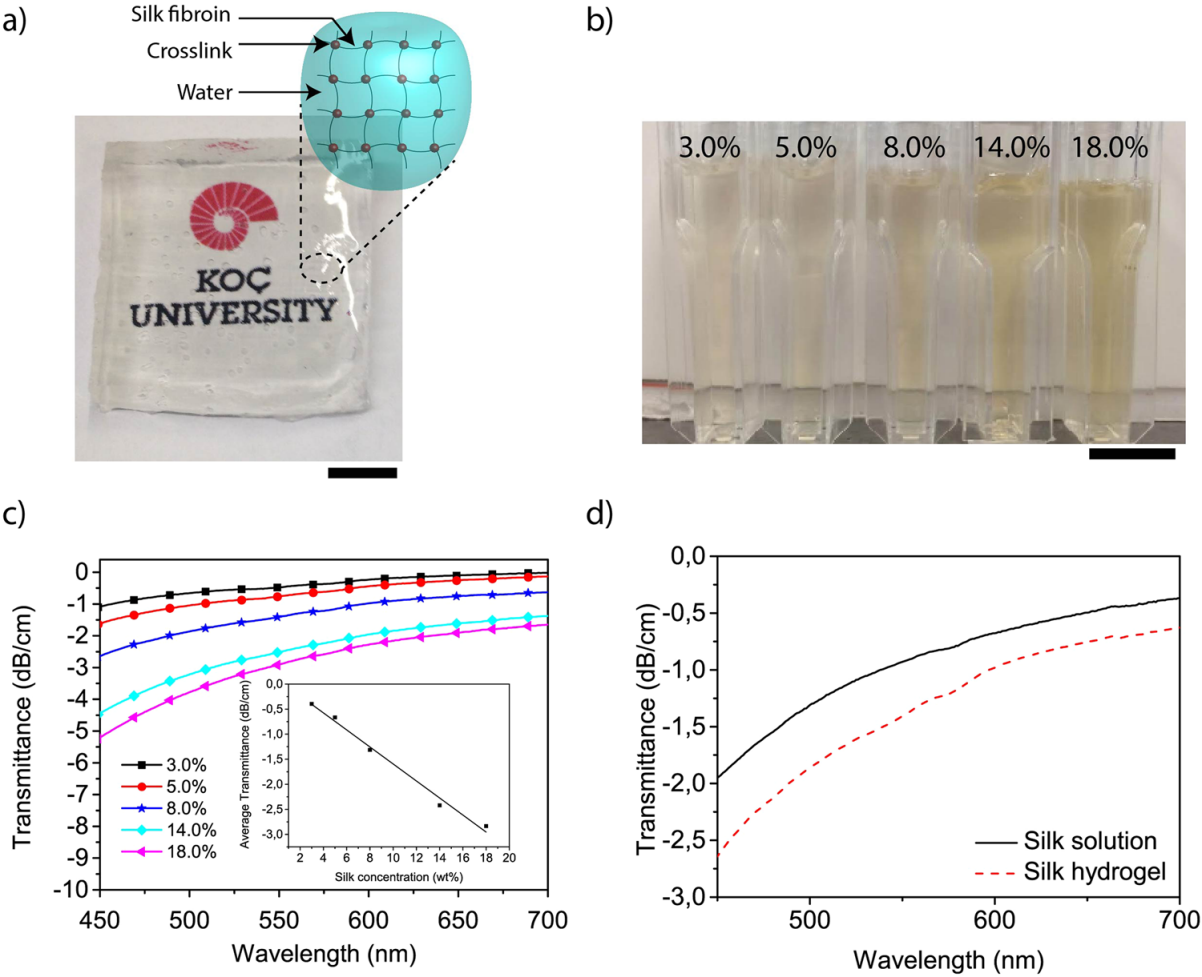
Optical properties of silk hydrogel. (**a**) Photograph showing the level of transparency of a silk hydrogel piece placed on top of the Koc University logo printed on a paper; inset: schematic of silk hydrogel structure showing water molecules that are trapped inside covalently crosslinked silk fibroin proteins. Scale bar, 0.5 cm. (**b**) Photograph of silk hydrogels with concentrations of 3.0, 5.0, 8.0, 14.0 and 18.0 wt%, respectively. Scale bar, 1 cm. (**c**) Transmittance of silk hydrogels in dB/cm units in visible spectrum at concentrations of 3.0, 5.0, 8.0, 14.0 and 18.0 wt%, respectively. Inset: Averaged transmittance of silk hydrogels in the visible spectrum. (**d**) Comparison of transmittance between the silk solution and hydrogel at the same concentration (8.0 wt%).

The optical transparency of materials is important for light guiding and extraction in optoelectronic devices^24^. Silk fibroin from *Bombyx mori* silkworm is a fibrous protein whose primary structure is dominated mainly by a repeating sequence of six aminoacids, (Gly-Ala-Gly-Ala-Gly-Ser)^25–27^. The silk fibroin is composed of two quasi-crystalline structures, silk I and silk II, with some amorphous residues in silk II (tyrosine)^28^. Each crystal site is statistically occupied by two antiparallel β-sheet chains with different relative orientations, the inter-sheet stacking occurring at a ratio of 1:2^29^. The quasi-crystalline structure of silk fibroin facilitates its use for optical applications in addition to structural, medical and tissue engineering applications^16–18^. Silk hydrogel can be chemically obtained by mixing silk fibroin solution with horseradish peroxidase (HRP) in the presence of hydrogen peroxide. The role of H_2_O_2_ is to activate the HRP by forming an oxyferryl center and a porphyrin-based cation radical at the active site of HRP, which makes the activated enzyme a powerful reducing agent^30^. HRP then undergoes two single electron oxidation reactions in the presence of a phenolic oxidizing agent (tyrosine in the silk fibroin) to return to its ground state. It produces two water molecules and two phenolic radicals of tyrosine which can react with each other to form covalent bonds. Based on this enzymatically catalysed reaction, we obtained a transparent network of cross-linked fibroin polymer chains containing a large amount of water (Fig. 1a).

We investigated the transmittance of hydrogel with the fibroin concentrations of 3.0, 5.0, 8.0, 14.0, and 18.0 wt% (Fig. 1b). In order to prepare silk hydrogels with lower and higher concentration, the initial fibroin solutions (8–10 wt%) were either diluted with pure water or concentrated by thermal evaporation. As the fibroin protein concentration increases, the silk hydrogels start to show a yellowish appearance due to high attenuation at shorter wavelengths (Fig. 1c). The attenuation of transmission was strongly dependent on fibroin concentration and wavelength. For example, 18 wt% fibroin hydrogel showed a −2.9 dB per cm at 550 nm (Fig. 1c), but as the protein content was decreased, the transmittance increased to −0.5 dB per cm at 550 nm for 3 wt% silk solution. Since the optical absorption of water is comparatively small (e.g., 0.0003 dB/cm at 550 nm)^31^, the loss of light in these hydrogels must be due to the silk proteins. In addition to the protein concentration, the crosslinking affected the optical properties. For this purpose we compared the change of the optical properties for the transition from silk solution to hydrogel phase (Fig. 1d). We observed that the average transmittance of hydrogels decreased approximately 0.41 dB/cm in the visible spectrum (from 450 to 700 nm) relative to the silk solution phase. Even though this effect was significant, it is relatively small in comparison with the concentration effect of silk fibroin proteins, which was −0.17 (dB/cm)/wt% (inset of Fig. 1c). Therefore, both the crosslinking process and protein concentration have direct effect on the optical properties of silk hydrogel.

The refractive index of silk hydrogel is another important parameter and the refractive index value needs to be slightly higher than the water due to its major water and the minor biopolymer content. The refractive index of silk hydrogel was calculated using equation 1, where C is the concentration of silk in solution (g/mL), dn/dC is the specific refractive index, and n_SH_ and n_water_ are the refractive index of silk hydrogel and water, respectively^32^. Here we know the specific refractive index of silk solution (dn/dC = 0.18 ml/g at 488 nm) and we obtained the refractive index of silk hydrogel as 1.35 at 488 nm. Moreover, the temperature (5–70 °C) does not significantly affect the optical performance of silk hydrogel^32^.

1 $$\frac{dn}{dC}=\frac{{n}_{SH}-{n}_{water}}{C}$$

### Intensity distribution of spatial radiation of silk hydrogel lenses

**Figure 2:**
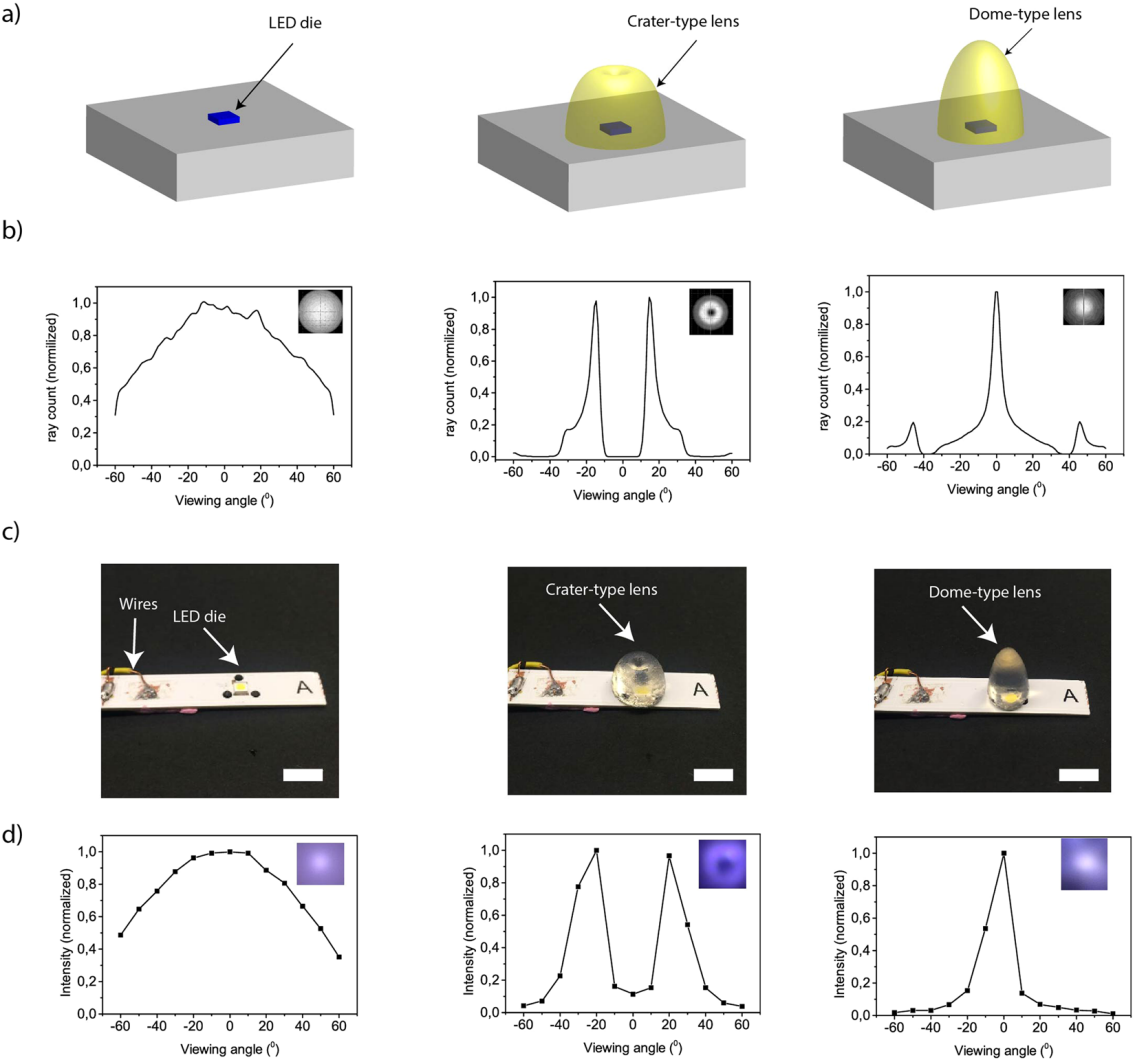
(**a**) Schematic representation of LED die (left), crater-type silk hydrogel lens on LED die (middle) and dome-type silk hydrogel lens on LED die (right). (**b**) Intensity distribution of spatial radiation of ray tracing simulation (without scattering effects). Inset: spatial radiation distribution. (**c**) Photographs of LED chip used for experimental testing (left), LED covered with crater-type hydrogel lens (middle) and dome-type hydrogel lens (right). Scale bar, 1 cm. (**d**) Experimental intensity distribution of spatial radiation of the LEDs. Inset: photographs of spatial radiation distribution.

To understand the spatial intensity distribution of LEDs with and without lens, we used ray tracing method. Light is generated via electroluminescence by the semiconductor die and refracted by the lens above the LED. We measured the radiation pattern of a LED die without a lens as the reference LED emission, and generated an identical LED die emission profile in the simulation. To understand the effect of lenses on light distribution profile, crater- and dome-type lenses^4^ were simulated on top of the LED die (Fig. 2a). Numerical simulation of ray tracing with and without lenses are shown in Fig. 2b. The crater-type lens (Fig. 2b, middle) exhibits maximum intensity of light at 16° and −16° which disperse light to wider angles (inset Fig. 2b, middle). In contrast, the dome-type lens (Fig. 2b, right) focuses light in the center (inset Fig. 2b, right).

To control the light intensity distribution we fabricated crater- and dome-type silk hydrogel lenses. An advantage of using hydrogels is that they can be simply molded into any shape required for specific optical application^12, 33–35^. To shape the silk hydrogels for lens application, the molds were designed (with Solidworks software) and 3D printed (with ULTRA 3SP) (Fig. S1). After the silk solutions were poured into their molds and covalently crosslinked, the solid structures were integrated on top of the dies (Fig. 2c). We experimentally measured the intensity distribution of spatial radiation of crater-type silk hydrogel lens and dome-type silk hydrogel lens on a cool white LED (Fig. 2d). The dome-type lens shows similar focusing of light around the center (inset Fig. 2d, right). The crater-type lens showed maximum intensity of light at 20° and −20° (inset Fig. 2d, middle), which is close to the value of numerical simulations. When we re-sampled simulation data with 10 degree polar angle intervals which was identical with the experimental sampling condition, the peak angle for crater- and dome-type lenses of simulation and experimental results were equal. However, it was observed that silk hydrogels broaden the peaks in crater- and dome-type lenses, probably due to scattering, (Fig. 2d) in comparison with the simulation (in which the scattering effect was not included) (Fig. 2b).

**Figure 3:**
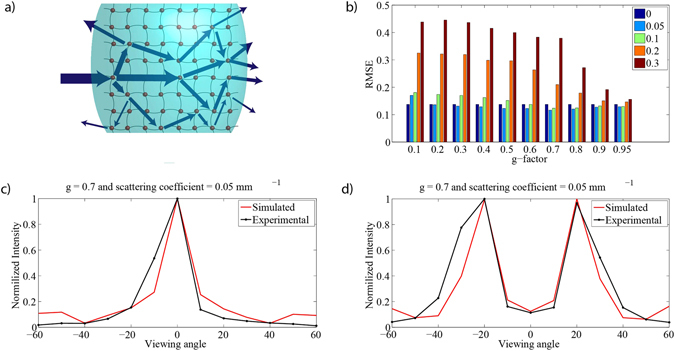
(**a**) Schematic representation of scattering in silk protein hydrogel. (**b**) Root mean square error between experimental and ray tracing results. Scattering coefficient was varied between 0 and 0.3 mm^−1^ and g-factor was varied between 0.1 and 0.95. (**c**) Experimental measurement and simulated ray tracing of intensity distribution of spatial radiation for dome-type lens and (**d**) for crater- type lens while minimum error was obtained at a scattering coefficient of 0.05 mm^−1^ and g-factor of 0.7.

To understand the scattering phenomena in silk protein hydrogels, we introduced scattering and anisotropy g-factor to the ray tracing simulation. When light is scattered, it deviates from its expected trajectory in a homogenous medium between two spatial points defined by the Fermat’s Principle of least time (Fig. 3a), and the amount of retained light after a single scattering event is defined by the anisotropy factor. The scattering in silk hydrogels originate due to the local refractive index changes by the fibroin protein and water. The scattering coefficients for the simulated hydrogels were swept between 0–0.3, and the anisotropy factors were swept between 0.1–0.95 both for crater- and dome-type lenses to understand the spatial broadening of light distribution. The intensity distribution of spatial radiation were obtained for each value of scattering coefficient and anisotropy factor, and sampled with 10° intervals. Then, the root mean square error (RMSE) between experimental and simulated data were calculated. The RMSE of dome- and crater-type errors between simulated and experimental data for each sweeping condition is shown in Fig. 3b. The minimum average error was obtained for a scattering coefficient of 0.05 per mm and an anisotropy factor of 0.7, which lead to a similar angular intensity profile for dome- and crater-type radiation patterns, respectively (Fig. 3c and d for dome- and crater-type lenses, respectively).

### Light extraction and stability of silk hydrogel lenses

**Figure 4:**
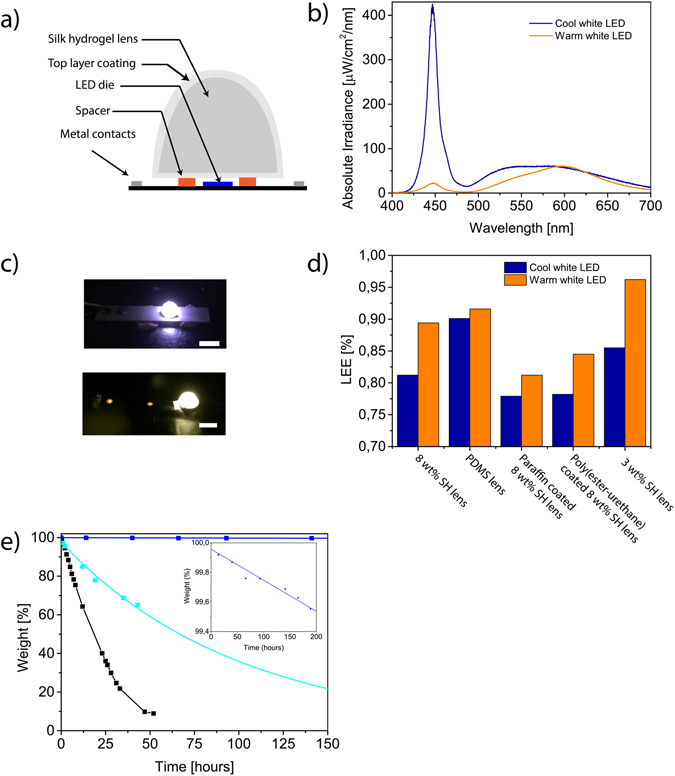
Light extraction efficiency and stability of biomaterial lenses. (**a**) Schematic of silk hydrogel with biopolymer layer coating. (**b**) Absolute irradiance of cool and warm white LED. (**c**) Photographs of 8 wt% silk hydrogel lens on a cool white LED (top) and on a warm white LED (bottom). Scale bar, 1 cm. (**d**) Light extraction efficiency (LEE) of silk hydrogel (SH) lenses with and without top coating, and PDMS lens on cool and warm white LEDs. (**e**) Weight decay of silk hydrogel lens. Black line: sole silk hydrogel lens, cyan line: 8 wt% silk hydrogel lens with poly(ester-urethane) coating, blue line: 8 wt% silk hydrogel lens with paraffin wax coating. Inset: zoomed weight decay of silk hydrogel lens with paraffin wax coating.

We investigated the optical efficiency of silk hydrogel lenses. To analyze the light extraction efficiency (LEE) of lenses, we prepared silk hydrogel hemisphere lenses (Fig. 4a) and measured LEE of the samples using an integrating sphere for white LEDs (Supplementary Fig. S2). Since the silk hydrogel transmittance was affected by the wavelength of the light, we tested the extraction efficiency for both cool and warm white LEDs, which have distinct blue and orange peaks in the visible spectrum (Fig. 4b and c). While the 8 wt% silk hydrogel lens had a LEE of 0.81 for a cool white LED (Supplementary Table S1), LEE of the lens increased to 0.89 for a warm white LED (Table 1). This increase in the efficiency from a cool to a warm white LED is expected due to the rise in the transmittance of the silk hydrogel at longer wavelengths (Fig. 1). Another important parameter that can enhance the extraction efficiency is the decrease of the silk concentration in the hydrogel. Thus, we decreased the silk concentration to 3 wt% and extraction efficiency dramatically increased to 0.85 for a cool white LED and 0.96 for a warm white LED (Fig. 4d). We compared the performance of the silk hydrogel with another widely used material of polydimethylsiloxane (PDMS) and the PDMS lens showed LEE of 0.90 for cool white LED and 0.91 for warm white LED. Therefore, silk hydrogel lenses with 3 wt% is preferable for the integration with a warm white source that can significantly enhance the performance of the power conversion efficiency of the LEDs.

**Table 1:** Optical parameters of biomaterial lenses on warm white LED. LEE: Light extraction efficiency, SH: silk hydrogel.

Lens	LER (lm/W_opt_)	Luminous flux (lm)	Luminous efficiency (lm/W_elec_)	Electrical input Power (mW)	Optical output Power (mW)	LEE	Chromaticity coordinates (x, y)
No lens	362	2.78	78.68	35.33	7.70	—	0.46, 0.42
8 wt% SH lens	365	2.51	71.04	35.33	6.89	0.89	0.47, 0.43
PDMS lens	366	2.58	73.02	35.33	7.06	0.91	0.46, 0.42
Paraffin wax coated 8 wt% SH lens	365	2.28	64.50	35.33	6.25	0.81	0.47, 0.43
Poly(ester-urethane) coated 8 wt% SH lens	372	2.42	68.49	35.33	6.51	0.84	0.47, 0.44
3 wt% SH lens	370	2.74	77.55	35.33	7.41	0.96	0.46, 0.43

We also studied the stability of silk hydrogel lenses by measuring its weight change due to the evaporation at 23 °C. The half lifetime of only silk hydrogel (without any polymer coating) was approximately 24 hours (Fig. 4e). To enhance the lifetime of silk hydrogel lenses, we synthesized biocompatible and biodegradable poly(ester-urethane) coating material by the stoichiometric reaction of 1,6-hexamethylene diisocyanate (HMDI) (Acros Organics, >99.5%) and polycaprolactone diol oligomer (PCL) (<M_n_> = 2000 g/mol)^36^, and coated the hydrogel lens with the poly(ester-urethane) film. The biopolymer film coating increased the lifetime approximately 3-fold to 70 hours. To further extend the lifetime, a film that shows a strong water vapour barrier needs to be incorporated on the hydrogel lens. For that paraffinic, wax-like materials show high performance since they can significantly block the moisture vapour transmission and they can even be obtained from natural sources (such as beeswax)^37^. We selected the use of paraffin wax, which is an edible biopolymer^37^ and used its film to coat the hydrogel. Thus, the lifetime has increased to 2 years and 341 days. Even though the top coating decreased the light extraction, other biopolymers such as polylactic acid, polymethyl methacrylate and waxes can be also explored to determine the coating layer for optimum light extraction efficiency and stability.

## Conclusions

In summary, the replacement of conventional plastics with eco-friendly materials is important for a sustainable and clean environment. The lenses occupy an important volume and mass in LEDs. As a solution, in this study we introduced the biomaterial of silk hydrogels as an optical material for lens application in LEDs. For this we extracted silk fibroin proteins from cocoons, transformed it into its hydrogels and fabricated crater- and dome-type lenses to control the spatial intensity profile. The scattering due to the proteins and crosslinks had direct effect on the optical properties. Silk hydrogel lenses showed light extraction efficiency over 0.95 on a warm white LED and the stability was significantly increased with a biocompatible and biodegradable poly(ester-urethane) coating. Moreover, using edible paraffin wax coating the stability of silk hydrogel lens was approximately increased by three orders of magnitude. Therefore, biomaterial lenses show promise for eco-friendly device applications.

## Methods

### Silk fibroin solution preparation

Silk solution preparation was carried out according to the ref. 38 as follows: 5 g cocoons were cut in half and the worms disposed of then boiled in 2 L, 0.02 M Na_2_CO_3_ (Sigma-Aldrich) solution for 30 mins to remove gum like sericin. Then, degummed cocoons were rinsed twice for 20 min in 1 L deionized water under continuous stirring and dried in air. Dried degummed cocoons were mixed with 9.3 M LiBr (Sigma-Aldrich) solution (1 g/4 ml) and kept in oven at 60 °C for 4 h. Dissolved silk was dialyzed with dialysis cassettes (3500 MWCO, Thermo Scientific) against 1 L deionized water for 2 days under continuous stirring to remove LiBr. Water was changed periodically at 1, 4, 12, 24 and 36 h. Finally, the silk solution was centrifuged at 9000 rpm for 20 min at −2 °C twice to remove impurities. The obtained silk solutions were 8–10 wt% in water. To obtain higher concentration of silk solution, 15 ml of 8–10 wt% silk solution was concentrated in oven for 4 h, at 60 °C. By thermal concentration, we achieved silk solution concentration of up to 28 wt%.

### Silk hydrogel preparation

1000 U/ml stock type VI horseradish peroxidase (HRP) (Sigma-Aldrich) solution were prepared by mixing 4 mg of HRP with 1 ml deionized water^17^. Silk hydrogels were prepared by adding 10 U of HRP to 1 ml of silk solution. After sonication, 10 µl of 1% H_2_O_2_ was added to 1 ml of silk HRP solution^21^. In 20 mins, silk hydrogels were formed.

### Transmittance Measurements

3.0, 5.0, 8.0, 14.0, 18.0 wt% silk solution was prepared by increasing or decreasing water content in 8 wt% silk solution. 2 ml silk hydrogel was prepared and inserted in 1 cm cuvettes to determine UV-Vis transmittance (Shimadzu UV-3600 - UV-VIS-NIR Spectrophotometer).

### Intensity distribution of spatial radiation measurements and ray tracing simulation

Silk hydrogel lens was mounted into LG Innotek WLED. 360° rotating platform was used with spectrophotometer (CCS 220, Thorlabs) to measure the optical intensity from WLED through silk hydrogel lens. Afterwards, TracePro60 ray tracing software were used. Crater- and dome-type shapes were generated in Solidworks software then inserted into TracePro60. Light generating source in TracePro60 was matched with LG Innotek WLED with uniform spatial profile and Gaussian angular profile with half angle of 90° in X and Y coordinates. 748501 rays at 546.1 nm was used. Then, scattering and anisotropy g-factor was introduced, Hunyey-Greenstein scattering model was used, in TracePro60 and radiation pattern was analyzed.

### Light extraction efficiency measurement

Silk hydrogel hemisphere lenses with 3 and 8 wt% silk solutions at a diameter of 7 mm were prepared and placed into an integrated sphere (Ocean Optics, FOIS-1). A spectrophotometer (Ocean Optics, Torus) connected integrating sphere and HL-3 VIS-NIR light source (Ocean Optics, HL-3 VIS-NIR) were used to measure the light extraction efficiency of the prepared samples. As control experiments, the reference light intensity was initially measured without any lens, and the extraction efficiency was calculated. Angle dependent measurements of silk hydrogel lenses were done using 360° rotating stage and spectrophotometer.

### Evaluation of silk lens stability

Using molds a silk hydrogel prepared and its weight was measured. For 2 days the decay in weight was measured. Afterwards, a silk hydrogel was prepared and a top layer of paraffin wax and poly(ester-urethane) was coated to increase its stability, and for 8 days the decay in weight was measured.

### Polymerization procedure

4.50 g (2.25 mmol) PCL and 0.38 g (2.25 mmol) HMDI were introduced into a 100 mL round bottom reaction flask, equipped with an overhead stirrer, nitrogen inlet and a reflux condenser. 9.0 g tetrahydrofuran (THF) (Aldrich > 99%) was added as the solvent and 50 ppm dibutyl tin dilaurate as catalyst and the system was heated to reflux. Completion of the reaction, which took about 2 hours, was determined by monitoring the disappearance of strong isocyanate absorption peak around 2270 cm^−1^ with an FTIR spectrophotometer. Number average molecular weight (45,000 g/mol) and polydispersity index (PDI = 1.63) of the polyester-urethane obtained was determined by size exclusion chromatography in THF, using polystyrene standards. Solvent cast films were strong and tough.

## Electronic supplementary material

Supporting Information

## References

[1] Kyle, B. New EPA Report Shows We are Generating More E-waste But Also Recycling More. http://www.electronicstakeback.com/2013/06/24/new-epa-report-shows-we-are-generating-more-e-waste-but-also-recycling-more (2013).

[2] Sesini, M. *The garbage patch in the oceans: the problem and possible solutions* Columbia University, New York (2011).

[3] Akasaki, I., H. Amano and S. Nakamura *The Nobel Prize in Physics 2014* (2014).

[4] Chien C-LC (2013). Polymer dispensing and embossing technology for the lens type LED packaging. Journal of Micromechanics and Microengineering.

[5] Irvine-Halliday, D., R. Peon, G. Doluweera, I. Platonova and G. Irvine-Halliday Solid-state lighting: the only solution for the developing world. *SPIE Newsroom*, 10(2.1200601**)**, p. 0056 (2006).

[6] Bergh A, Craford G, Duggal A, Haitz R (2001). The promise and challenge of solid-state lighting. Physics today.

[7] Moran, B. Light-Emitting Diodes (LEDs) for Lighting Applications. *BCC Research paper* (2014).

[8] Schubert, E.F., Gessmann, T. and Kim, J. K. *Light emitting diodes 193* (Wiley Online Library, 2005).

[9] Eberspacher, C. and Fthenakis, V. M. Disposal and recycling of end-of-life PV modules. In Photovoltaic Specialists Conference, *Conference Record of the Twenty-Sixth IEEE*. IEEE (1997).

[10] Irimia-Vladu M (2014). “Green” electronics: biodegradable and biocompatible materials and devices for sustainable future. Chemical Society Reviews.

[11] Hoffman AS (2012). Hydrogels for biomedical applications. Advanced drug delivery reviews.

[12] Choi M (2013). Light-guiding hydrogels for cell-based sensing and optogenetic synthesis *in vivo*. Nature photonics.

[13] Min B-M (2004). Electrospinning of silk fibroin nanofibers and its effect on the adhesion and spreading of normal human keratinocytes and fibroblasts *in vitro*. Biomaterials.

[14] Motta A (2004). Fibroin hydrogels for biomedical applications: preparation, characterization and *in vitro* cell culture studies. Journal of biomaterials science, Polymer edition.

[15] Murphy AR, Kaplan DL (2009). Biomedical applications of chemically-modified silk fibroin. Journal of materials chemistry.

[16] Nicolson PC, Vogt J (2001). Soft contact lens polymers: an evolution. Biomaterials.

[17] Partlow BP (2014). Highly tunable elastomeric silk biomaterials. Advanced functional materials.

[18] Mitropoulos AN (2015). Transparent, nanostructured silk fibroin hydrogels with tunable mechanical properties. ACS Biomaterials Science & Engineering.

[19] Zhao S (2016). Bio-functionalized silk hydrogel microfluidic systems. Biomaterials.

[20] Applegate, M.B., C. Alonzo, I. Georgakoudi, D.L. Kaplan and F.G. Omenetto, A simple model of multiphoton micromachining in silk hydrogels. *Applied Physics Letters*, **108**(24) (2016)

[21] Applegate MB (2015). Laser-based three-dimensional multiscale micropatterning of biocompatible hydrogels for customized tissue engineering scaffolds. Proceedings of the National Academy of Sciences.

[22] Zhang DY, Lien V, Berdichevsky Y, Choi J, Lo YH (2003). Fluidic adaptive lens with high focal length tunability. Applied Physics Letters.

[23] Sher CW (2016). A high quality liquid-type quantum dot white light-emitting diode. Nanoscale.

[24] Nizamoglu, S. *et al*. Bioabsorbable polymer optical waveguides for deep-tissue photomedicine. *Nature communications* **7** (2016). 10.1038/ncomms10374 PMC4735646 26783091

[25] Asakura T, Watanabe Y, Itoh T (1984). NMR of Silk Fibroin. 3. Assignment of Carbonyl Carbon Resonances and Their Dependence on Sequence and Conformation in Bombyx mori Silk Fibroin Using Selective Isotopic Labeling. Macromolecules..

[26] Asakura T, Yoshimizu H, Yoshizawa Y (1988). NMR of Silk Fibroin. 9. Sequence and Conformation Analyses of the Silk Fibroins from Bombyx mori and Philosamia Cynthia ricini by I5N NMR Spectroscopy. Macromolecules.

[27] Asakura, T.; Kaplan, D. L. Encyclopedia of Agricultural Science (ed. Arutzen, C. J.,) 1-11 (Academic Press: New York, Vol. 4, 1994).

[28] Asakura T, Kuzuhara A, Tabeta R, Saitô H (1985). Conformation Characterization of Bombyx mori Silk Fibroin in the Solid State by High-Frequency 13C Cross Polarization-Magic Angle Spinning NMR, X-ray Diffraction, and Infrared Spectroscopy. Macromolecules..

[29] Takahashi Y, Gehoh M, Yuzuriha K (1999). Structure refinement and diffuse streak scattering of silk (Bombyx mori). Int. J. Biol. Macromolecules.

[30] Rodríguez-López N (2001). Mechanism of Reaction of Hydrogen Peroxide with Horseradish Peroxidase: Identification of Intermediates in the Catalytic Cycle. J. Am. Chem. Soc..

[31] Venyaminov SY, Prendergast FG (1997). Water (H 2 O and D 2 O) molar absorptivity in the 1000–4000 cm− 1 range and quantitative infrared spectroscopy of aqueous solutions. Analytical biochemistry..

[32] Nagarkar S, Nicolai T, Chassenieux C, Lele A (2010). Structure and gelation mechanism of silk hydrogels. Physical Chemistry Chemical Physics.

[33] Peppas NA, Hilt JZ, Khademhosseini A, Langer R (2006). Hydrogels in biology and medicine: from molecular principles to bionanotechnology. Advanced Materials.

[34] Yeh J (2006). Micromolding of shape-controlled, harvestable cell-laden hydrogels. Biomaterials..

[35] Dong L, Agarwal AK, Beebe DJ, Jiang H (2006). Adaptive liquid microlenses activated by stimuli-responsive hydrogels. Nature.

[36] Yilgor I, Yilgor E (2007). Structure‐Morphology‐Property Behavior of Segmented Thermoplastic Polyurethanes and Polyureas Prepared without Chain Extenders. Polymer Reviews..

[37] Bourtoom T (2008). Edible films and coatings: characteristics and properties. International Food Research Journal.

[38] Rockwood DN (2011). Materials fabrication from Bombyx mori silk fibroin. Nature protocols..

