# Supplementary material for: Silk-hydrogel Lenses for Light-emitting Diodes
Source: Sci Rep. 2017 Aug 3;7:7258. doi: 10.1038/s41598-017-07817-1 (PMC5543068; doi:10.1038/s41598-017-07817-1)
Supplement: Supplementary file 1 — Supporting Information [file 41598_2017_7817_MOESM1_ESM.pdf]

Supporting Information

Silk-hydrogel Lenses for Light-emitting Diodes

Rustamzhon Melikov[1], Daniel Aaron Press[1], Baskaran Ganesh Kumar[1], Itir Bakis Dogru[2], Sadra Sadeghi[3], Mariana Chirea[1], İskender Yılgör[4], and Sedat Nizamoglu[1,2,3]

[1]Department of Electrical and Electronics Engineering, Koc University 34450 Sariyer, Istanbul, Turkey.
[2]Graduate School of Biomedical Sciences and Engineering, Koc University 34450 Sariyer, Istanbul, Turkey.
[3]Graduate School of Materials Science and Engineering, Koc University 34450 Sariyer, Istanbul, Turkey.
[4]Department of Chemistry, Koc University 34450 Sariyer, Istanbul, Turkey.

E-mail: snizamoglu@ku.edu.tr

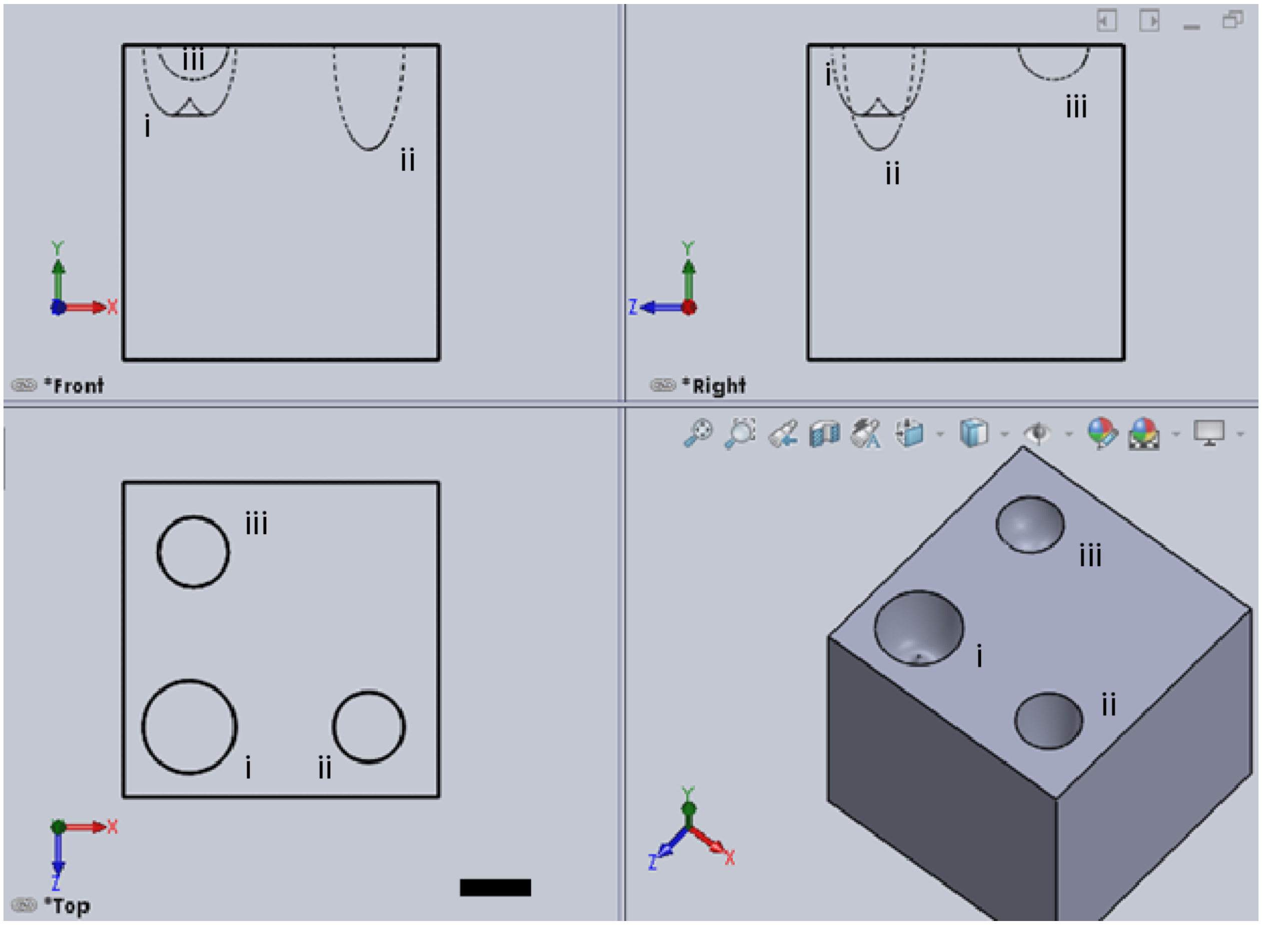

Figure S1. Mold structure to prepare silk hydrogel lenses. Scale bar, 1 cm. i: crater-type, ii: dome-type and iii: hemispherical lens structures.

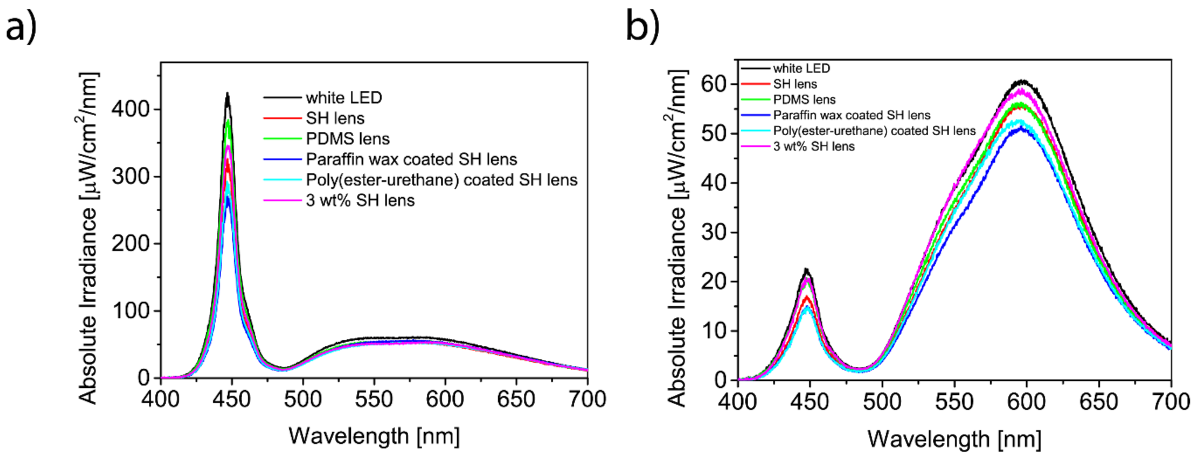

Figure S2. Absolute irradiance of silk hydrogel lenses with and without top coats and PDMS lens on a a) cool white LED and b) warm white LED.

Table S1. Optical parameters of biomaterial lenses on cool white LED. LEE: Light extraction efficiency, SH: silk hydrogel.

Lens	LER (lm/W_{opt})	Luminous flux (lm)	LE (lm/W_{elec})	Electrical input power (mW)	Optical output power (mW)	LEE (%)	Chromaticity coordinates (x,y)
No lens	227	3.96	150.2	26.36	17.47	-	0.27,0.22
8 wt% SH lens	239	3.39	130.4	26.36	14.19	0.81	0.29,0.24
PDMS lens	226	3.56	135.1	26.36	15.74	0.90	0.27,0.22
Paraffin wax coated 8 wt% SH lens	257	3.49	132.4	26.36	13.61	0.77	0.30,0.26
Poly(ester-urethane) coated 8 wt% SH lens	246	3.35	127.1	26.36	13.66	0.78	0.29,0.25
3 wt% SH lens	230	3.44	130.5	26.36	14.94	0.85	0.28,0.23
